# Intravenous enoxaparin as alternative ECMO anticoagulation over a period of 94 days: a case report

**DOI:** 10.1186/s13019-023-02226-0

**Published:** 2023-04-11

**Authors:** Miroslav Durila, Jan Berousek, Vlasta Vlasakova, Tomas Vymazal

**Affiliations:** Department of Anaesthesiology and Intensive Care Medicine, Second Faculty of Medicine, Charles University and Motol University Hospital, V Uvalu 84, 150 06 Prague 5, Czech Republic

**Keywords:** ECMO, Extracorporeal membrane oxygenation, Enoxaparin, LMWH, PFA 200, Primary haemostasis

## Abstract

**Background:**

Unfractionated heparin is used worldwide as a standard anticoagulation therapy for extracorporeal membrane oxygenation (ECMO) machines. However, its use brings about significant bleeding and thrombotic complications for critically ill patients. This case report shows that low molecular weight heparin together with ECMO-produced primary haemostasis pathology can be used as an alternative way of ECMO anticoagulation.

**Case presentation:**

This paper presents the case of a patient with respiratory failure who subsequently suffered from cardiac failure and spent 94 days on combined V-V and V-A ECMO devices (two ECMO devices running simultaneously on one patient) with intravenous enoxaparin used instead of unfractionated heparin anticoagulation. No life-threatening bleeding/thrombotic events happened during this period, nor did any technical problems with ECMO occur.

**Conclusions:**

In this case report, continuous intravenous low molecular weight heparin anticoagulation was used as a safe alternative to ECMO anticoagulation.

## Background

Extracorporeal membrane oxygenation (ECMO) is a very useful technique used for organ support in patients with refractory respiratory or cardiac failure. To prevent ECMO circuits from clotting, unfractionated heparin (UFH) is used most of the time as standard anticoagulation worldwide [1]. However, ECMO machines produce complex coagulopathy that consists of a pathology of secondary haemostasis (fibrinolysis, coagulation factor deficiency, thrombocytopenia, heparin overdose) as well as primary haemostasis pathology (acquired von Willebrand syndrome, acquired platelet dysfunction) [2]. We have recently published two studies describing primary haemostasis pathology found in patients on ECMO support as detected by a PFA 200 analyser and Multiplate aggregometry [3, 4], and we showed that together with low molecular weight heparin (LMWH), this combination may prevent ECMO devices and the patients themselves from clotting [3]. This paper presents the case of a patient who spent 94 days on ECMO without life threatening bleeding or thrombotic complications. He only received continuous intravenous enoxaparin anticoagulation, as primary haemostasis pathology was detected by a PFA 200.

## Case presentation

This is the case of a 43-year-old man who spent 10 days in a small hospital on mechanical ventilation because of pneumonia brought on by COVID-19. Because his condition had deteriorated due to a bacterial superinfection (Moraxella catarrhalis, Haemophilus influenzae and Staphylococcus aureus), he was indicated for ECMO organ support and was transferred to our hospital. Immediately after being admitted, V-V ECMO (veno-venous ECMO) was implanted via his right femoral vein (inflow cannula 23 Fr, this size of cannula was chosen because the diameter of the femoral vein was 9 mm) and his right jugular vein (outflow cannula 23 Fr), and 6000 IU of enoxaparin was administered as bolus i.v. before ECMO canulation. This was followed by the continuous intravenous infusion of enoxaparin, aiming for higher prophylactic anti Xa – 0.4–0.6 IU/ml (the initial speed of the infusion was 600 IU/hour, adjusted according to the anti Xa level - measured every 8 h) according to the local protocol. He was connected to ECMO (a Cardiohelp machine was used with a blood flow of 4.4 L/min and 3000 rpm), immediately after which his blood gases significantly improved and protective artificial ventilation was achieved.

**Fig. 1:**
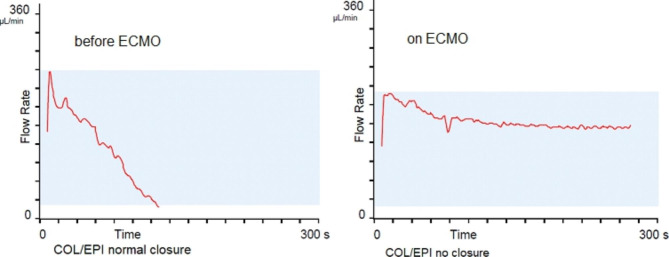
Primary haemostasis evaluated by a PFA 200 COL/EPI before ECMO implantation and on ECMO

**Fig. 2:**
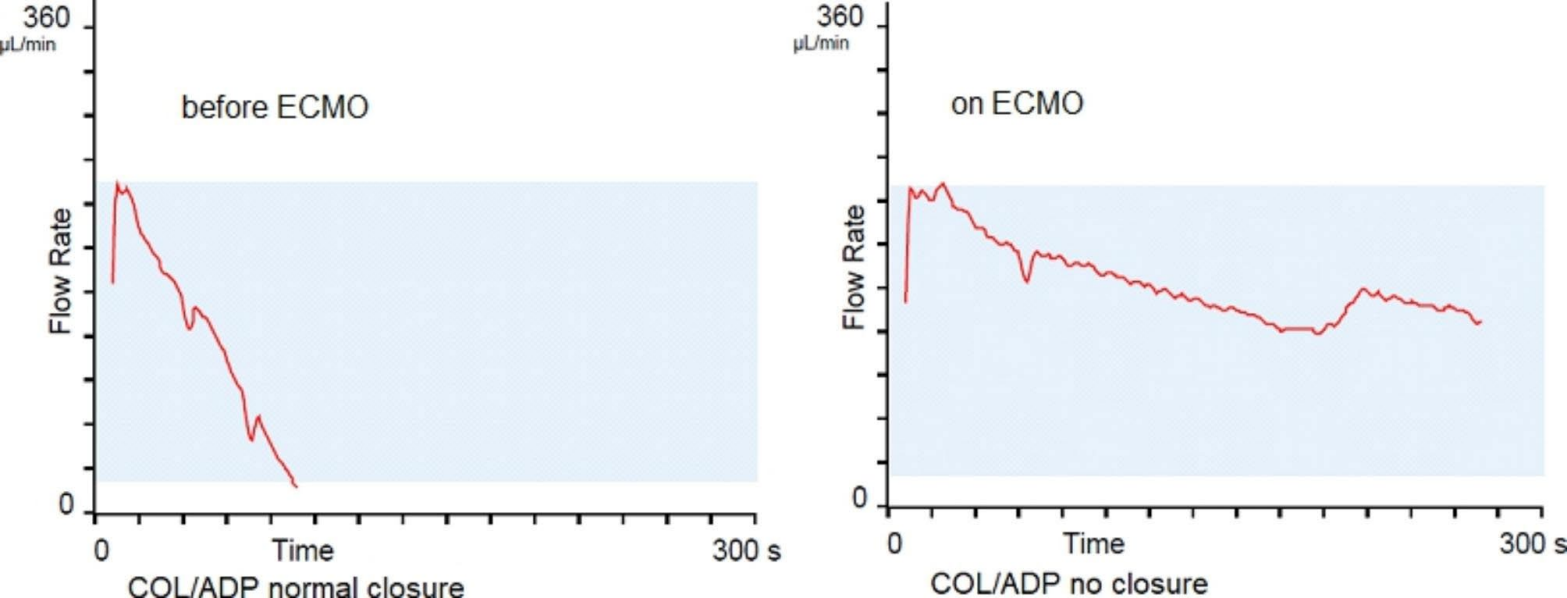
Primary haemostasis evaluated by a PFA 200 COL/ADP before ECMO implantation and on ECMO

The situation worsened on the 5th day of hospitalisation when spontaneous pneumothorax developed, which was treated with drainage. Additionally, a small bleeding in the respiratory tract occurred that was caused by the pathology in primary haemostasis detected by a PFA 200 COL/EPI and COL/ADP with a picture of “no closure” (Figs. 1 and 2).

.

There was no pathology of secondary haemostasis evaluated by ROTEM, and anti Xa was at an acceptable level at 0.5 IU/ml. Bleeding was successfully treated with von Willebrand factor containing drugs and 1 mg of recombinant activated FVII (FVIIa, to bypass primary haemostasis). In the following days after decreasing the level of sedation, the patient started to interfere with the artificial ventilation, causing technical problems for the ECMO machine (the presence of intermittent high negative pressure at inflow cannula caused by the cannula sucking onto the surface of the inferior vena cava, IVC). Therefore, on the 8th day of hospitalisation, V-V ECMO was converted to VV-V ECMO (inflow cannula was added via left femoral vein 19 Fr). His blood was drained from the distal part of the IVC via the left femoral vein and the superior vena cava (SVC) via the right jugular vein, and the returning blood went into the proximal part of the IVC via the right femoral vein. With the VV-V ECMO setup, it was possible to warrant uninterrupted blood flow from the ECMO machine and oxygen delivery to the body (blood flow 5.5 L/min, 3500 rpm), decrease sedation and start physiotherapy with the patient. On day 10, a surgical tracheostomy was performed without bleeding complications or an interruption of the enoxaparin infusion. On day 44, VV-V ECMO was reduced to V-V ECMO (the same setup as at the beginning), as lung function and patient cooperation improved slightly (the cannula was removed from the left femoral vein). To maximise the physiotherapy potential, a 27 Fr Avalon ECMO cannula was introduced on day 51 via the left jugular vein, (the old cannulas from the right femoral and jugular veins were removed). However, the situation worsened again because of another pulmonary infection accompanied by right heart failure (we detected pulmonary hypertension with right heart dilatation without pulmonary embolism on a CT scan). Therefore, on the 57th day, another V-A ECMO machine (veno-arterial ECMO) was implanted via the left femoral vein and artery (with distal perfusion cannula), and the patient was connected to two separate ECMO machines for another 8 days with only enoxaparin anticoagulation with anti Xa 0.5-0.6IU/ml. The reason this approach was chosen over other ECMO configurations such as V-VA was to prevent very high rounds per minute and the flow on the ECMO device (having implanted Avalon cannula), which would represent a higher risk and lead to the disturbance of platelets, erythrocytes and other blood components.

Over the next 8 days, he recovered from heart failure and V-A ECMO was surgically explanted without any thrombus in the arterial system of the femoral artery. In the following days, his lung function also improved, and the patient was weaned from V-V ECMO on day 94. Beginning that same day, an intravenous continuous infusion of enoxaparin was replaced with a subcutaneous form of enoxaparin, meaning 2 × 40 mg s.c. every 12 h. On day 112, the patient’s lung function improved. Not only was he successfully weaned from artificial ventilation, the tracheostomy cannula was removed as well. The patient was transferred back to the original hospital on his 118th day in our department. He did not develop any major bleeding or thrombosis complications that would require invasive intervention during the entire period, nor did the ECMO machine ever stop unexpectedly. A very small bleeding from the respiratory tract was successfully treated with appropriate drugs. Throughout the whole period, the ECMO set (oxygenator) had to be changed out 10 times (meaning the ECMO oxygenator had to be replaced every 9 days on average) because laboratory markers of hemolysis and coagulopathy were present (an especially high level of fibrin monomers > 150 mg/L and D-dimmers > 15000ug/L, a decrease in the fibrinogen level).

## Discussion

The ECMO machine is a very useful device used for heart/lung support, and continuous intravenous unfractionated heparin (UFH) is used worldwide as standard ECMO anticoagulation to prevent clotting in the device [1]. Recent studies by Jose I Nunez et al., who analysed the data of V-V ECMO patients from the Extracorporeal Life Support Organization registry, found that 40.2% of these patients experienced ≥ 1 bleeding and thrombosis event (thrombotic events comprised 54.9% of all the cases and were predominantly ECMO circuit thrombosis), and the inpatient mortality rate of those patients was 34.9% [5]. Similarly, Mabel Chung et al. analysed V-A ECMO patients from the Extracorporeal Life Support Organization registry and found that 44.1% of those patients suffered some kind of hemocompatibility-related adverse events (62.1% bleeding, 37.9% thrombotic events and the crude in-hospital mortality rate was 58.6%) [6]. We recently published two studies describing primary haemostasis pathology found in patients on ECMO support that was detected by PFA 200 analysers and Multiplate aggregometry [3, 4], and we showed that this phenomenon can be used together with LMWH as an alternative way of ECMO and patient anticoagulation [3]. We use this combination routinely, and this case supports our previous findings. The ECMO oxygenator itself represents a high shear stress system, and this environment predisposes it to increased platelets activation via adhesion and aggregation leading to platelet clot formation. Histological analyses of ECMO thrombus formation show that despite UFH use, ECMO thrombus consists of von Willebrand factor (vWF), platelets, red blood cells, leukocytes and fibrin. This finding supports the dominating role of primary haemostasis taking place on the surface of oxygenators, subsequently leading to the consumption of relevant components and their dysfunction. Developed primary haemostasis pathology in combination with LMWH might protect the ECMO device and the patient against clotting.

Compared to UFH, LMWH does not inhibit thrombin formation (it does not prolong thrombin time). Therefore, procoagulant therapy can be more efficient when a patient is on LMWH compared to UFH (vWF containing drugs, activated factor seven, fibrinogen) in case of bleeding. Interestingly, Gao et al. demonstrated that despite its anticoagulation effect on fibrin formation, unfractionated heparin may have an activating effect on platelet clot formation by promoting platelet responsiveness via its ability to initiate αIIbβ3-mediated outside-in signaling [7]. That would at least partially explain the phenomenon of why thrombotic complications occur so often despite UFH anticoagulation (especially thrombosis in ECMO devices) [5]. The different pharmacodynamics of UFH and LMWH may justify using LMWH from the very beginning of ECMO implantation rather than using it as a secondary option.

One of the disadvantages of LMWH might be its relatively long half-time and the absence of a highly effective antidote, although protamine can be used in case of heavy bleeding or surgery. Frequently monitoring anti-Xa and keeping it strictly within the low range of 0.4–0.6 IU/ml is critical, and despite lacking data, this level seems to be safe to prevent bleeding. One could speculate or disagree that changing the ECMO set relatively often based on significant laboratory pathology might explain the absence of haemostatic complications. We have to admit that this might be another factor preventing haemostatic complications. Therefore, the daily assessment of laboratory tests plays an important role in guiding the decision process regarding when ECMO sets should be changed.

## Conclusion

Our case demonstrates that ECMO organ support using enoxaparin might represent a safe alternative to unfractionated heparin for various types of ECMO support, even for a period as long as 94 days.

## Data Availability

As this paper is a case report, all generated and analyzed data are included in this article.

## List of abbreviations

COL/EPI: collagen/epinephrine
COL/ADP: collagen/adenosine diphosphate
CT: computerized tomography
ECMO: extracorporeal membrane oxygenation
IU: international units
LMWH: low molecular weight heparin
PFA: platelet function analyzer
ROTEM: rotational thrombelastometry
IU: international units
UFH: unfractionated heparin
